# Genome-Wide Characterization of Selection Signatures and Runs of Homozygosity in Ugandan Goat Breeds

**DOI:** 10.3389/fgene.2018.00318

**Published:** 2018-08-14

**Authors:** Robert B. Onzima, Maulik R. Upadhyay, Harmen P. Doekes, Luiz. F. Brito, Mirte Bosse, Egbert Kanis, Martien A. M. Groenen, Richard P. M. A. Crooijmans

**Affiliations:** ^1^Animal Breeding and Genomics, Wageningen University and Research, Wageningen, Netherlands; ^2^National Agricultural Research Organization (NARO), Entebbe, Uganda; ^3^Department of Animal Breeding and Genetics, Swedish University of Agricultural Sciences, Uppsala, Sweden; ^4^Department of Animal Biosciences, Centre for Genetic Improvement of Livestock (CGIL), University of Guelph, Guelph, ON, Canada

**Keywords:** *Capra hircus*, homozygosity, adaptation, genomic inbreeding, genetic diversity, selective sweeps, candidate genes

## Abstract

Both natural and artificial selection are among the main driving forces shaping genetic variation across the genome of livestock species. Selection typically leaves signatures in the genome, which are often characterized by high genetic differentiation across breeds and/or a strong reduction in genetic diversity in regions associated with traits under intense selection pressure. In this study, we evaluated selection signatures and genomic inbreeding coefficients, *F*_ROH_, based on runs of homozygosity (ROH), in six Ugandan goat breeds: Boer (*n* = 13), and the indigenous breeds Karamojong (*n* = 15), Kigezi (*n* = 29), Mubende (*n* = 29), Small East African (*n* = 29), and Sebei (*n* = 29). After genotyping quality control, 45,294 autosomal single nucleotide polymorphisms (SNPs) remained for further analyses. A total of 394 and 6 breed-specific putative selection signatures were identified across all breeds, based on marker-specific fixation index (*F*_ST_-values) and haplotype differentiation (hapFLK), respectively. These regions were enriched with genes involved in signaling pathways associated directly or indirectly with environmental adaptation, such as immune response (e.g., *IL10RB* and *IL23A*), growth and fatty acid composition (e.g., *FGF9* and *IGF1*), and thermo-tolerance (e.g., *MTOR* and *MAPK3*). The study revealed little overlap between breeds in genomic regions under selection and generally did not display the typical classic selection signatures as expected due to the complex nature of the traits. In the Boer breed, candidate genes associated with production traits, such as body size and growth (e.g., *GJB2* and *GJA3*) were also identified. Furthermore, analysis of ROH in indigenous goat breeds showed very low levels of genomic inbreeding (with the mean *F*_ROH_ per breed ranging from 0.8% to 2.4%), as compared to higher inbreeding in Boer (mean *F*_ROH_ = 13.8%). Short ROH were more frequent than long ROH, except in Karamojong, providing insight in the developmental history of these goat breeds. This study provides insights into the effects of long-term selection in Boer and indigenous Ugandan goat breeds, which are relevant for implementation of breeding programs and conservation of genetic resources, as well as their sustainable use and management.

## Introduction

Goats are among the most important livestock species in developing countries, such as Uganda, playing a significant socio-economic, nutritional and cultural role in smallholder production systems (MAAIF, 2011). The total goat population in Uganda is estimated to consist of over14 million animals, predominantly from indigenous breeds (98%) and a small proportion (2%) from exotic breeds (MAAIF and UBOS, 2009; UBOS, 2015). Exotic breeds have been artificially selected for production traits over several generations, whereas indigenous breeds have undergone no or less intense artificial selection. While exotic breeds are subjected to more intense artificial selection, it is expected that the effect of natural selection (i.e., adaptation to the specific environment) is more apparent in the indigenous breeds and has played an important role in their development. Based on this hypothesis, it is expected that indigenous breeds will tend to exhibit resistance to gastro-intestinal parasites and local diseases, tolerance to heat, water scarcity and ability to use low quality fodder. Often high order traits like adaptation to environmental stress are influenced by several traits acting in combination. Adaptation is a complex trait that involves many biological processes and quantitative trait loci with each having a small but cumulative effect on the overall expression of the phenotype (Kim et al., 2016; Yang et al., 2016; Mwacharo et al., 2017).

Selection (both natural and artificial) is one of the main driving forces shaping genetic variation across genomes of livestock species. Under strong positive selection pressure, the frequency of favorable alleles will increase over time (Maynard and Haigh, 2007). This may result in genomic regions with high genetic differentiation across breeds and/or specific haplotypes rising to high frequencies. Such regions can thus be selection signatures. Analysis of selection signatures has the goal of identifying genomic regions or loci showing deviations from neutrality. Other forces like migration, admixture events, and population bottlenecks may have a profound effect on genomic variability, locally increasing or reducing the genetic variation.

Two well-established methods to detect selection signatures include the fixation index (*F*_ST_) (Wright, 1949; Weir and Cockerham, 1984; Porto-Neto et al., 2013) and haplotype differentiation statistic – hapFLK (Fariello et al., 2013). *F*_ST_ is one of the most popular methods to detect selection signatures if data is available for multiple populations. The *F*_ST_-approach measures population differentiation due to locus-specific allele frequencies between populations and can detect highly differentiated alleles undergoing divergent selection among populations (McRae et al., 2014; Zhao et al., 2015). A drawback of the approach is that the *F*_ST_-statistic assumes that all populations are of similar effective population size and are derived independently from the same ancestral population. The hapFLK - statistic measures differences of haplotype frequencies between populations and accounts for the hierarchical structure of the populations (Fariello et al., 2013). The use of a combination of haplotype information and of the hierarchical structure of populations results in greater power for the detection of selection signatures.

Selection signature analyses using genome-wide SNPs have been widely applied in exploring the genomes of livestock species such as sheep (Kijas et al., 2012; Purfield et al., 2017; Rochus et al., 2018), cattle (Porto-Neto et al., 2014; Zhao et al., 2015; Taye et al., 2017), and goats (Burren et al., 2016; Kim et al., 2016; Brito et al., 2017). These studies have identified genes associated with a variety of traits including thermo-tolerance, immune response, reproduction functions, skin and hair structure, feed intake, and metabolism.

Ugandan indigenous goat breeds can be phenotypically categorized within three main breeds: Kigezi, Mubende, and Small East African (Mason and Maule, 1960). Other indistinct ecotypes of indigenous goat breeds also exist including Karamojong and Sebei (Nsubuga, 1996). These breeds show high genetic diversity, but weak population sub-structuring (Onzima et al., 2018). The result of the weak population structure is low levels of inbreeding and some of the breeds having similar selection signatures (Msalya et al., 2017). The indigenous breeds present a high degree of adaptation to parasites and heat tolerance, and survive on poor quality fodder, while also maintaining good reproductive rates (Mwacharo et al., 2017). However, production levels are much lower compared to specialized breeds. Therefore, Boer goats were introduced in Uganda in the early 1990s to genetically improve the growth rate and body size of the indigenous breeds (Nsubuga, 1996). Because of community-based small ruminant breeding programs and the use of limited Boer breeding males for cross breeding, the increase in inbreeding levels is a major concern to the industry.

The increase in inbreeding in livestock at a genomic level over generations leads to a reduction in genetic diversity. When an offspring is inbred, it may inherit autozygous chromosomal segments from both parents that are identical by descent (IBD), i.e., segments that are derived from a common ancestor (Broman and Weber, 1999). The result is continuous homozygous segments in the genome, also known as runs of homozygosity (ROH). The extent of ROH can be used to estimate the inbreeding coefficient (Bosse et al., 2012; Marras et al., 2015; Peripolli et al., 2018). ROH can be used to disclose the genetic relationships among individuals, usually estimating with high accuracy the autozygosity at an individual and/or population levels (Ferenčaković et al., 2011, 2013a). It can also be used to establish the level of selection pressure on the populations (Zhang et al., 2015). Length and frequency of ROHs may also be used to distinguish distant from more recent inbreeding, since the length of IBD segments follows an inverse exponential distribution with a mean of 1/2 *g* Morgans, where *g* is the number of generations from a common ancestor (Howrigan et al., 2011).

The objectives of this study were to: (1) identify unique selection signatures in the genome and the genes under selection in Ugandan goat breeds, and (2) assess the occurrence and distribution of ROH and ROH-based genomic inbreeding in Ugandan goat breeds.

## Materials and Methods

### Animals and Genotype Quality Control

The data used in this study were derived from 144 animals from 6 goat populations and has been described in detail previously (Onzima et al., 2018). The animals were from the five indigenous breeds, Mubende (*n* = 29), Kigezi (*n* = 29), Small East African (*n* = 29), Karamojong (*n* = 15) and Sebei (*n* = 29), and from the exotic Boer breed (*n* = 13). All animals were genotyped with the Illumina GoatSNP50 BeadChip (Tosser-Klopp et al., 2014), which features 53,347 single nucleotide polymorphisms (SNPs). Genotype quality control (QC) procedures were performed using PLINK v1.90 (Chang et al., 2015). All samples passed the quality criteria (missing genotype call rate ≥ 0.1) and were used in the analysis. The SNPs with a call rate below 0.95, a minor allele frequency (MAF) lower than 0.05, located on non-autosomal chromosomes, or not in Hardy Weinberg Equilibrium (at *p* < 0.001) were discarded. After QC procedures, 46,105 autosomal SNPs remained. For these SNPs, the position on the genome was obtained from the goat reference genome assembly ARS1 release 102 (Bickhart et al., 2017). After removing SNPs with unknown position on the ARS1 genome assembly, 45,294 autosomal SNPs from 144 goats remained in the final dataset.

### Relatedness Within and Between Breeds

The level of relatedness between individuals (both within and between breeds) was determined using genomic similarities. For each pair of individuals, the genomic similarity (SIM_SNP_*ij*__) was determined according to Malécot (1948):

$$SIM_{\text{SNP}}{}_{{}_{ij}}=\frac{\sum_{k=1}^{n_{\text{SNP}}}\left(I_{11,k}+I_{12,k}+I_{21,k}+I_{22,k}\right)}{4n_{\text{SNP}}}$$

where *n_SNP_* is the total number of markers and *I_xy,k_* is an indicator variable that was set to 1 when allele *x* of individual *i* and allele *y* of individual *j* at marker *k* were identical by state (IBS), and to 0 otherwise. Note that, as self-similarities were included, the average similarity in a breed was equivalent to the expected homozygosity in that breed.

### Identifying Selection Signatures

To increase the likelihood to detect true selection signatures (i.e., no false positive results), multiple approaches can be used (Simianer, 2014). The methods adapted for analysis of selection signatures need to be robust enough to disentangle selective pressures from other effects on the population such as migration, admixture and population bottlenecks. In this study, we used allele specific population differentiation defined as *F*_ST_ (Wright, 1949) and a haplotype-based differentiation approach, hapFLK (Fariello et al., 2013), which accounts for haplotype structure of populations and for variable effective population sizes.

#### Fixation Index (*F*_ST_)

Selection signatures for each breed were identified using an *F*_ST_-statistic per SNP that compares the allele frequency in the breed to the allele frequency in a combined population of the remaining breeds, following the unbiased estimator proposed by Weir and Cockerham (1984) and implemented in PLINK (Chang et al., 2015). For example, differences between the exotic Boer breed on the one hand, and the indigenous goat breeds on the other, were investigated by calculating the *F*_ST_-values between Boer and a combined population of all the indigenous breeds. We also computed *F*_ST_-values for the indigenous breeds while excluding Boer from the analysis. However, as the exclusion of Boer did not influence the results for the indigenous breeds, only results with Boer included are reported in the subsequent sections.

In general, genomic regions showing high *F*_ST_-values with moving averages (mas) and single SNPs indicate strong breed differentiation or selection, while low *F*_ST_-values suggest no or a limited amount of population differentiation. Negative *F*_ST_-values were set to zero, as they imply no genetic differentiation between the two groups.

To visualize and infer region-specific differences over the erratic pattern of individual SNPs, we computed a ma of *F*_ST_ (*maF*_ST_) values for 5 adjacent SNPs. The *maF*_ST_ was computed for 5 adjacent SNPs and plotted against the chromosomal position for all goat autosomes (CHI coordinates). The SNPs with a *maF*_ST_ above the 95% quantile of the empirical distribution of raw *F*_ST_-values were considered as putative selection signatures. The ma is a simple approach for identifying regions of interest in the genome from the erratic pattern of SNPs. This approach has been implemented successfully in analyzing systematic differences in response to genetic variation to pedigree and genome-based selection methods in chicken (Heidaritabar et al., 2014) and genome-wide genetic diversity in Dutch dairy cattle (Doekes et al., 2018). By using *maF*_ST_, rather than *F*_ST_ for single SNPs, we aimed to reduce the influence of the small sample sizes on the results.

#### Haplotype Differentiation (hapFLK)

To account for haplotype structure and varying effective population sizes, we used hapFLK (Fariello et al., 2013) to detect potential selection signatures in the six goat populations. The used procedure has been described in detail by Brito et al., (2017). Briefly, hapFLK was applied to the unphased genotype data to identify putative regions under selection, by estimating the neighbor joining tree and a kinship relationship matrix based on Reynolds’ genetic distances between the breeds. The pairwise Reynolds’ distances (Reynolds et al., 1983) between populations (including an outgroup) are computed for each SNP and averaged over the genome. Using the genotype data and kinship matrix, and assuming 6 clusters in the fastPHASE model (-k, 6), the program was run and the hapFLK statistic was computed as an average of 20 expectation maximization iterations to fit the Linkage Disequilibrium (LD) model. With hapFLK values generated for each SNP, *p*-values were computed based on a chi-square distribution of the numerical values. The mean and variance of hapFLK distribution were estimated and used to standardize each SNP specific value. This was subsequently followed by computation of *p*-values from a standard normal distribution, and the (-log10) of *p*-values was plotted against the genomic positions. To minimize the number of false positives, a *q*-value threshold of 0.01 was set to control the false discovery rate (FDR) at the 1% level. Putative selective signatures were defined by the regions with a threshold of *p* < 0.005.

### Identification of Candidate Genes Associated With Selection Signatures

Genes within putative selection signature regions were retrieved from NCBI^1^, using the goat reference assembly ARS1. The genes overlapping either partially or fully within the 95% threshold of the empirical distribution of the raw *F*_ST_ -values and within the regions with *p* < 0.005 for hapFLK, were putative selection candidate genes.

For each of the breeds, gene enrichment analyses were performed based on the *F*_ST_, and hapFLK results with the web-based tool, Database for Annotation, Visualization, and Integrated Discovery (DAVID) v6.8 (Huang et al., 2009; Jiao et al., 2012), which allows for the investigation of the Kyoto Encyclopedia of Genes and Genomes (KEGG) pathways (Kanehisa et al., 2012) and Gene Ontology (GO) for biological processes (Ashburner et al., 2000). Fisher’s exact test (*p*-value = 0.05), was applied to identify significantly enriched GO biological and functional processes. More stringent settings, such as Bonferroni correction, FDR, and Fold enrichment test were not considered in the detection given the limited scope of the study. Human gene ontologies were used since the goat genome has not been properly annotated. Moreover, the human genome is highly annotated than closely related species like bovine; thereby increasing the probability of retrieving GO terms in the goat genome. Phenotypes known to be affected by the identified candidate genes were compared from literature and using the AnimalQTLdb at: https://www.animalgenome.org/cgi-bin/QTLdb/index.

### Runs of Homozygosity (ROH) and Genomic Inbreeding (*F*_ROH_)

For each individual, ROHs were identified using an in-house script which incorporates a set of criteria for defining regions of homozygosity.

An ROH was called if the following criteria were fulfilled: (1) 20 or more consecutive SNPs were homozygous, (2) a minimum physical length of 2 Mb, (3) a maximum gap between two consecutive SNPs of 500 Kb, and (4) maximum of 2 missing genotypes and no heterozygous calls within ROH. The rather stringent criteria were used to minimize incorrect discovery of ROH (false positives) within regions of low marker density. The minimum expected length of homozygous DNA segments was based on the time frame of approximately 25 generations, over which goats are believed to have been characterized in separate breeds in Uganda (Mason and Maule, 1960). The length of ROH derived from a common ancestor *g* generations ago follows an inverse exponential distribution with the mean equal to 100/2 *g* cM (Fisher, 1954; Thompson, 2013). A genetic distance of approximately 1 cM per Mb is often assumed in cattle (Arias et al., 2009) and assuming a similar relationship for goats, the mean length of ROH derived from common ancestor from 25 generations ago would be 2 Mb.

The proportion of ROH per animal in comparison to the whole genome SNP coverage provides a useful indication of the level of inbreeding. Genomic inbreeding coefficient based on ROHs were computed as the length of the autosome covered by ROHs divided by the overall length of the autosome covered by the SNPs (McQuillan et al., 2008):

$$F_{\text{ROH},i}=\frac{L_{\text{ROH}}}{L_{\text{AUTO}}}$$

where *L*_ROH_ is the sum of the total length of ROH in individual *i* and *L_AUTO_* is the total length of the autosomes covered by the SNPs (2.463 Gb). The number of ROHs and *F*_ROH_ were also evaluated for different ROH length categories. We focused on length classes from 2 to 16 Mb to investigate more ancient inbreeding (2 and 16 Mb are the expected lengths of ROH derived from common ancestors 25 and 3 generations ago, respectively) and >16 Mb to assess more recent inbreeding (expected length of ROH derived from ancestors ≤ 3 generations ago).

The ROH were estimated in each individual separately and then classified into four length categories: 2–4 Mb, 4–8 Mb, 8–16 Mb, and >16 Mb, following classification used in similar studies (Kirin et al., 2010; Ferenčaković et al., 2013a; Marras et al., 2015), specified from now on as ROH_2-4__Mb_, ROH_4-8__Mb_, ROH_8-16__Mb_, and ROH_>__16__Mb_, respectively. For each length category in each of the individuals of each breed, we computed the total number of ROH identified – nROH, mean sum of ROH coverage – *S*_ROH_ in Mb (defined by sum of all ROH per individual divided by the number of animals per breed) and average length of ROH (*L*_ROH_, Mb).

## Results

### Relatedness Among Ugandan Goats

Mean genomic similarities within and between breeds are shown in **Table 1**. As expected, within breed similarities (diagonal) were higher than between breed similarities (off-diagonal). Within breeds, the highest mean similarity was found in Kigezi (0.643) and the lowest in Sebei (0.623). Between breeds, the indigenous Ugandan goat breeds showed higher genomic similarity with each other than the Boer breed.

**Table 1:** Mean genomic similarity within (diagonal) and between (off-diagonal) six goat breeds.

	BOE	KIG	MUB	SEA	KAR	SEB
BOE	0.625	–	–	–	–	–
KIG	0.551	0.643	–	–	–	–
MUB	0.553	0.622	0.625	–	–	–
SEA	0.549	0.615	0.614	0.632	–	–
KAR	0.550	0.607	0.607	0.606	0.627	–
SEB	0.549	0.613	0.611	0.610	0.610	0.623

*BOE = Boer (n = 13), KIG = Kigezi (n = 29), MUB = Mubende (n = 29), SEA = Small East African (n = 29), KAR = Karamojong (n = 15), and SEB = Sebei (n = 29).*

### Selection Signatures

#### Selection Signatures – *F*_ST_

There was generally a high level of differentiation between Boer on the one hand, and the indigenous Ugandan breeds on the other. For Boer, the average *F*_ST_ across all SNPs was 0.123, while the average *F*_ST_ for the indigenous breeds was less than 0.050 (**Supplementary Table S1**).

**FIGURE 1:**
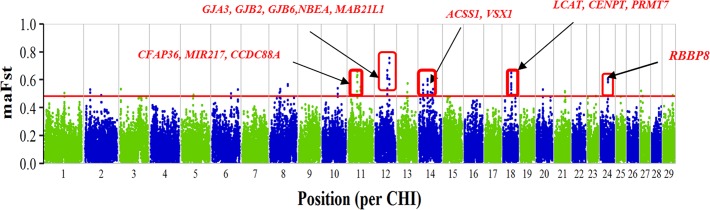
Plot of moving average *F_ST_*-values of SNPs per chromosome. The plot shows comparison of Boer goats with other Ugandan goat breeds and candidate genes in putative selective signatures are shown in red. The horizontal red line (at 0.482) represents the 95%-percentile of raw *F_ST_*-values.

Analysis of breed specific differentiation between each of the Ugandan goat breeds including the Boer breed resulted in several putative regions of selection as shown in **Supplementary Table S2** (*p* < 0.05, without Bonferroni correction). In Boer, the 29 putative regions of selection were identified, which spread across 17 autosomes and overlapped with 134 genes. The regions with the highest degree of differentiation were found on CHI11, 12, 14, 18, and 24 (**Figure 1**). The highest ranked SNP window (*maF*_ST_-value = 0.754) was found on CHI12 in a genomic region between 60.170 and 60.711 Mb and overlap with portions of the genes *MAB21L1* and *NBEA.* Analysis of breed specific differentiation for the Ugandan indigenous goat breeds resulted in 394 putative regions of selection distributed across all the breeds showing some candidate genes for traits of economic importance. The regions varied from 66 in Mubende to 79 in the Small East African goats distributed across most of the autosomes (**Supplementary Table S2**). The selection signature regions in the indigenous goat breeds are on average numerous and shorter than in the Boer. There is limited overlap between the different breeds indicating signatures are mostly breed specific. Several genes were found spanning the selection signature regions across the autosomes (**Supplementary Table S2**). Functional analysis of some of the candidate genes (**Supplementary Figure S1**) shows they may be involved in tropical adaptation such as thermo-tolerance and immune response in the indigenous breeds. These include *KPNA4* (CHI1), *MTOR* (CHI16), *SH2B1* (CHI25), and *MAPK3* (CHI25) in Karamojong; *IL10RB*, *IFNAR*, *DNAJC13* (CHI1) in Kigezi; *PPP1R36* and *HSPA2* (CHI10), *DNAJC24* (CHI5) in Mubende; *CD80*, *ADPRH*, *IGSF11* (CHI1), *IGF1* (CHI5) in Small East African goats, and *HOXC12* and *HOXC13* (CHI5) in Sebei. The full gene-list is found in **Supplementary Table S2**.

#### Selection Signatures – hapFLK

**FIGURE 2:**
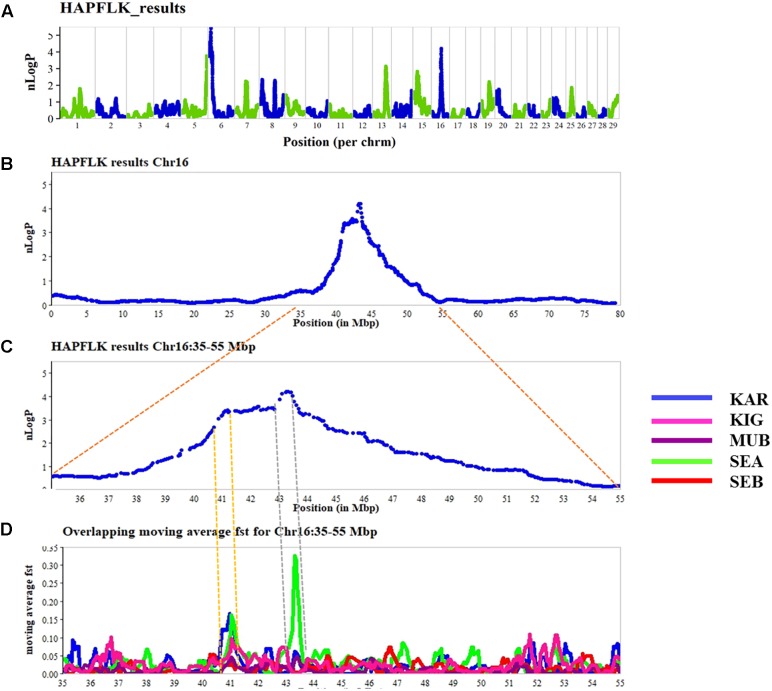
Genome-wide scan for putative signatures of selection using the hapFLK **(A)**. The negative log of *p*-values is plotted in chromosomal order. The alternating colors green and blue represent the different chromosomes (on the upper panel). On the lower panels, **(B–D)** is a zoom into the chromosome 16 to show the regions overlapping with high *F_ST_* and hapFLK values, defining putative selection signatures over four selected regions in the different breeds (KAR = Karamojong, KIG = Kigezi, MUB = Mubende, SEA = Small East African, and SEB = Sebei).

The results of the haplotype-based differentiation with hapFLK are shown in **Figure 2**. A significance threshold of *p* < 0.005 was considered to identify regions under selection. The hapFLK analysis resulted in six putative selection signature regions on CHI5 (116.662–118.773 Mb), CHI6 (0.005–16.337 Mb), CHI8 (7.766–7.941 Mb), CHI13 (58.709–63.989 Mb), CHI15 (14.932–23.571 Mb), and CHI16 (40.533–45.988 Mb). Some of the candidate genes identified, which may be playing a role in tropical adaption include; *CFI* (CHI 6), *DEFB* genes (CHI 13), and *ASIP* (CHI 13), *MTOR*, *PIK3CD* (CHI 16), and *CD44* (CHI 15) (**Supplementary Table S3**).

Four of the six significant regions identified by hapFLK partially overlapped with the 394 significant selective signature regions identified by *F*_ST_. Several short overlapping regions were found between hapFLK and *F*_ST_, with the strongest signals detected on CHI 6, 13, and 16 (**Figure 2**). Some of the regions were breed specific and contained several genes (**Supplementary Table S4**).

### Gene Enrichment of Putative Selection Signatures

Within the putative selection regions identified, a list of genes was identified for each of the approaches used: *F*_ST_ (**Supplementary Table S2**) and hapFLK (**Supplementary Table S3**) and were used to perform separate functional analyses using DAVID with default settings on the human gene set (Huang et al., 2009; Jiao et al., 2012). Functional analysis of the *F*_ST_ gene-list for each of the breeds yielded 47 significant (*p* < 0.05) gene ontology (GO) biological process (BP) terms (**Supplementary Table S5**) and 15 KEGG pathways were enriched (**Supplementary Table S6**). The biological processes enriched were related to cell communication, male sex differentiation, microtubule cytoskeleton organization in the Boer; and negative regulation of catalytic activity, homeostasis in number of cells within the tissue, TIR-domain-containing adapter-inducing interferon-β (TRIF)-dependent toll-like receptor signaling pathway (GO:0035666), positive regulation of peptidyl-tyrosine phosphorylation (GO:0050731), cytokinesis (GO:0000910), and angiogenesis (GO:0001525) among others in the indigenous goat breeds.

The DAVID analyses based on hapFLK gene list across the breeds resulted in 18 significant (*p* < 0.05) biological processes (**Supplementary Table S5**) and nine significant (*p* < 0.05) KEGG pathways (**Supplementary Table S6**). The genes identified, were significantly involved in the defense response to bacterium (GO:0042742; *p*-value < 0.001), negative regulation of the apoptotic process (GO:0043066; *p*-value < 0.001), and positive regulation of gene expression (GO:0010628; *p*-value < 0.001) among others.

### Analysis of ROH and Genomic Inbreeding – *F*_ROH_

**FIGURE 3:**
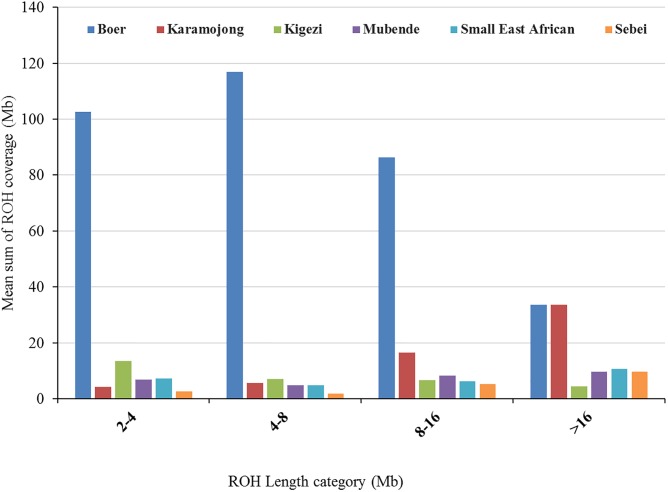
Genome-wide distribution of mean sum of ROH coverage per length category averaged per breed across six Ugandan goat breeds.

A total of 1,497 ROHs were detected across all individuals. The frequency of ROHs and their length-distribution differed across breeds (**Figure 3**). For all length categories, ROHs were generally more frequent in Boer (a breed selected for meat production) than in Ugandan indigenous goat breeds. Consequently, Boer showed the highest genomic inbreeding coefficients (**Table 2**). For example, the mean *F*_ROH_ _≥2Mb_ in Boer was 13.8%, while for the indigenous breeds, it ranged from 0.8% (Sebei) to 2.4% (Karamojong). Shorter ROH were more frequent than longer ROH in all breeds except for Karamojong. In the later breed, there were remarkably many ROH > 16 Mb.

**Table 2:** Average percentage genomic inbreeding coefficient (*F*_ROH_) for different length categories of ROH across six goat populations.

Length category (Mb)	Boer (*n* = 13)	Karamojong (*n* = 15)	Kigezi (*n* = 29)	Mubende (*n* = 29)	SEA (*n* = 29)	Sebei (*n* = 29)
*N*_0_	0	2	1	5	2	13
*F*_ROH(2-4)_	4.17	0.17	0.54	0.28	0.30	0.11
*F*_ROH(4-8)_	4.75	0.23	0.28	0.20	0.20	0.08
*F*_ROH(8-16)_	3.50	0.67	0.27	0.33	0.26	0.21
*F*_ROH(>16)_	1.37	1.36	0.18	0.40	0.43	0.39
Total *F*_ROH≥2_ _Mb_	13.79	2.44	1.27	1.20	1.18	0.78

*SEA = Small East African goat, N_0_ = Number of individuals from the samples where no ROH ≥ 2 Mb, homozygous segments were detected, F_ROH__≥2Mb_ = overall percentage genomic inbreeding at ROH threshold of 2 Mb, F_ROH(x-y)_ = genomic inbreeding based on ROHs of length x–y Mb.*

The ROHs were located across the whole genome, with some regions showing a higher frequency than other (**Supplementary Figure S2**). The mean sum of ROH segment coverage was generally higher for short ROHs than for long ROHs. The highest mean ROH coverage within the short ROH category (ROH of 2–4 Mb) was found in Boer, while Sebei had the lowest of mean ROH coverage. For instance, around 65% of the Boer mean sum of ROH segment coverage in this study (219.65 Mb) was within the shorter ROH category 2–8 Mb. However, for the other breeds, the coverage ranged from 4.52 Mb in Sebei to 20.38 Mb in Kigezi (**Supplementary Table S7**). In the long ROH category (ROH_>__16__Mb_), Boer and Karamojong showed higher ROH genome coverage (33.67 and 33.60 Mb, respectively), which indicates more recent inbreeding. In the remaining breeds, the coverage was between 4.44 Mb in Kigezi and 10.58 Mb in the Small East African breed. Boer showed high genome coverage with both short and long ROH, suggesting that the breed has experienced both recent and ancestral inbreeding compared to Ugandan indigenous breeds. The findings also suggest that among the Ugandan indigenous goat breeds, Karamojong has greater levels of inbreeding compared to the others. The proportion of the genome located on an ROH differed between breeds and chromosomes. The proportion ranged from 1.50% on CHI2 to 93.62% on CHI23 in Sebei (**Supplementary Table S8**). ROH segments were identified on all 29 autosomes in Boer, but the number varied in the genomes of the indigenous goat breeds with several autosomes showing no homozygous regions (**Supplementary Table S9**).

## Discussion

In this study, we unravel selection signatures and genomic inbreeding coefficients in goat breeds of Uganda using genome-wide SNP data.

Various approaches have been implemented for the detection of selection signatures in several domestic animal species such as, cattle (Msalya et al., 2017; Taye et al., 2017), horses (Petersen et al., 2013), sheep (Kijas et al., 2012; Fariello et al., 2014; Rochus et al., 2018), and goats (Kim et al., 2016; Wang et al., 2016; Brito et al., 2017). In this study, we assessed the genome-wide differences between Ugandan indigenous goat breeds (Karamojong, Kigezi, Mubende, Small East African, and Sebei) and exotic Boer goats by using population differentiation, *F*_ST_ (Weir and Cockerham, 1984) and the haplotype structure in the populations, hapFLK (Fariello et al., 2013).

The statistical power to detect selection signatures may vary among the approaches. In this study, we used *F*_ST_ and hapFLK for detecting selection signatures. The use of different methods in detecting selection signatures boosts the accuracy of detection and eliminates unknown bias (Simianer, 2014; Ma et al., 2015).

### Selection Signatures

The genomic regions potentially under selection identified in this study spanned a myriad of candidate genes with diverse biological, molecular, and cellular functions, which could be because the adaptation processes to environmental stressors is controlled by a complex network of genes acting together, other than single candidate genes. For instance, adaptation to hot and arid environments was found to be mediated by a complex network of genes in Egyptian Barki goats and sheep (Kim et al., 2016), which were directly or indirectly associated with energy and digestive metabolism, autoimmunity, thermo-tolerance (melano-genesis) and, muscular and embryonic development. Similarly, adaptation may also result from interaction of several traits under the influence of several genes (Lv et al., 2014). In this study, we found putative signatures containing a complex of genes involved directly or indirectly in immune response. Moreover, selection for complex traits may also leave limited or none of classic selection signatures due to weak selection acting on the genome (Kemper et al., 2014).

In line with expected selection signatures for such complex traits, the genomic regions identified in this study using genome-wide maker specific fixation index in the populations showed limited overlap. This suggests that the selection on genes involved in adaptation to a tropical environment were breed-specific. Moreover, the selection signatures found in our study do not display classic hard sweep characteristics, which is to be expected for complex traits. This is in contrast to the findings with Valdostana goats in Italy (Talenti et al., 2017). This may arise due to the very diverse nature of the populations and absence of hard and long selection signature regions observed within the populations at the 50K SNP marker density. Second, our study pooled genotypes from six different breeds and lending to picking out differences between the breeds, unlike in the study of Talenti et al. (2017), whose focus was on only one breed.

We did find overlap between the selected regions identified with the hapFLK method and breed-specific *F*_ST_ signatures. Since hapFLK considers population stratification, the haplotypes in these regions are likely to be selected for in the corresponding breeds. The fact that these regions stand out in the hapFLK results as well as the *F*_ST_ results suggests that selection on those regions most closely resembles classic sweeps. Strong selection signatures were observed on CHI 6, 13, and 16, and they harbored several genes which may be important for adaptation in tropical environments, such as *MTOR* which is involved in heat stress response and the heat shock family of genes (Shi and Manley, 2007) and *DNAJC24* involved in the first apoptosis signal (FAS) pathway and regulation of stress induction of heat shock protein (hsp) in *Bos taurus* (Roy and Collier, 2012). Several of the genes are involved in immune response particularly the innate immune response pathway (GO:004508). Overall, several of the genes identified in this study are associated with tropical adaptation. Moreover, in the Boer, several candidate genes identified in the putative selection signatures are involved in production related traits, reflecting a more modern selection regime. However, to pin-point the exact genes involved in tropical adaptation and production in the Ugandan goat breeds, there is need for an in-depth study at high resolution.

Generally, most of the regions under selection were subtle and breed-specific, as expected for complex traits under selection. Therefore, the forces driving selection in the genome of the indigenous goat breeds in this study may be associated with adaptation to African tropical environment, such as: thermo-tolerance, disease and parasite resistance, and the ability to perform under limited (quality and quantity) feed and water resources. The genome-wide scans identified candidate genes within the putative selective signatures associated with specific biological pathways and functions, which may be shaping the genomic architecture of Ugandan goat breeds for survival in stressful environment. Although most signatures were breed-specific, some interesting similarities could be found in the adaptive processes the genes in selected regions were involved in.

#### Thermo-Tolerance Genes

Several candidate genes were identified, which are associated with adaptation to thermal stress. The homeobox genes *HOXC12* and *HOXC13* genes identified in Sebei are involved in the anterior/posterior pattern specification (GO:0009952). The genes play a role in hair follicle differentiation, growth, and development by regulating the keratin differentiation-specific genes (Wu et al., 2009; Taye et al., 2017). The *HOXC13* gene has been reported to influence skin thickness. Skin thickness and number of hair follicles impacts positively on thermoregulation. For instance, in cattle, thicker skin is associated with thermo-tolerant cattle (*Bos indicus*) as opposed to heat susceptible cattle (*Bos taurus*) breeds (Alfonzo et al., 2016). Relatedly, *PPP1R36* and Heat Shock Protein A2 (*HSPA2*) (CHI10, 26.402–26.719 Mb) identified in Mubende are involved in heat stress response and, *HSPA2*, *DNAJC24*, and *DNAJC13* are associated with the heat shock family of genes (Shi and Manley, 2007). The presence of multiple genes associated with heat stress would seem to suggest that the trait is under intense selection pressure in tropically adapted breeds. Genes such as *KPNA4* (CHI1), *MTOR* (CHI16), *SH2B1* (CHI25), and *MAPK3* (CHI25) were also identified in Karamojong goats. They have been reported to be involved in the FAS pathway and regulation of stress induction of hsp in *Bos taurus* (Roy and Collier, 2012). Furthermore, we identified the gene *IGF1* (CHI5, 64.576–65.310 Mb). *IGF1* encodes a protein that is similar to insulin and it is involved in regulation of carbohydrate and lipid metabolism. *IGF1* facilitates post-absorptive nutrient partitioning during heat stress and accumulation of insulin is often an adaptation mechanism to heat stress (Sanz Fernandez et al., 2015).

#### Adaptive and Innate Immunity Genes

Several candidate genes in the putative selection regions are involved in regulating innate and adaptive immunity in mammals. For example, we identified diacylglycerol kinase beta, *DGKB* gene in Small East African (CHI4 position 97.794–97.991 Mb). The gene is involved in the glycerolipid, glycerophospholipid, and phosphatidylinositol metabolic pathways and has been found to be associated with QTL for strongyles that includes *Haemonchus sp* (Zvinorova, 2017). Other candidate genes identified include *IL10RB* and *IFNLR1* on CHI1(0.693–0.959 Mb) in Kigezi goats. These genes are involved in type III Interferon Signaling Pathway and confer immunity (Ferrao et al., 2016). Similarly, we also identified candidate genes in Sebei such as *BCL2L1* (CHI13, 60.489–60.748 Mb), and in Small East African goats such as *ERBB2* (CHI19, 39.703–40.129 Mb), and *ENO1* (CHI16, 43.006–43.669 Mb). These genes are directly or indirectly associated with immunoregulation, e.g., *ENO1* in humans (Ryans et al., 2017).

The identification of cytokines such as *IL17RE*, *IL17RC*, and *IL23A* in this study may be associated with gastrointestinal parasite resistance. Some of the cytokines have been reported to be significantly upregulated in *Haemonchus contortus* infected sheep and are known to be involved in adaptive immune response (GO:0002250) (Guo et al., 2016). These results would suggest that immunity genes are hotspots for natural selection in Ugandan goat breeds in response to high burden of pathogen/parasite challenge in the local environment (Thumbi et al., 2014; Bahbahani and Hanotte, 2015). Indigenous goat breeds vary in the degree of response to parasite infestation (Chiejina and Behnke, 2011; Onzima et al., 2017). We hypothesize that the variation between the breeds may be due to the genes allowing for selection on resistance traits either naturally or artificially.

One of the regions in Boer on CHI3 (84.128–84.373 Mb) harbors a gene *PRMT6*, which is reported to influence early embryonic development in Zebra fish (Zhao et al., 2016). The gene *VAV3* (CHI3, 84.730–84.962 Mb) is also associated with the immune system (Shen et al., 2017). Interleukin 12A (*IL12A*) gene is another cytokine that was identified on CHI1 in Karamojong which may be associated with immune response. The gene family is reported to be involved with the immune system in humans through series of biological processes (Reitberger et al., 2017). Moreover, it is cytokine that acts as a growth factor for activated T and Natural Killer (NK) cells, enhances the NK/lymphokine activated killer cells and stimulates the production of IFN-gamma.

#### Genes Associated With Production Traits

The candidate gene *NBEA* (Neurobeachin) in the region on CHI12 (maFST = 0.754) (**Supplementary Table S2**) is associated with human body weight (Fox et al., 2007). Another gene of interest that we identified is *VAV3* (CHI3; 84.730–84.962 Mb) on a homozygous region in Boer. The gene has been identified as a candidate gene for efficiency of food conversion in swine (Wang et al., 2015) and in goats (Brito et al., 2017). These genes are particularly significant to be identified in Boer goats, which have been extensively selected for high body weight and growth rate. Earlier studies have also identified this gene as a top candidate in Draa goats in Morocco (Benjelloun et al., 2015). However, in that study it was not conclusive if the candidate gene was associated with body weight.

Other genes identified in Boer such as the gap junction protein genes *GJB2*, *GJB6*, and *GJA3* belong to the family of genes involved in cell communication (GO:0007154). They encode proteins that influence body size, skeletal and embryonic development and testicular embryogenesis, and may indirectly influence traits such as growth (Kim et al., 2016). The region (41.943–42.086 Mb) on CHI13 contains another gene *ACSS1* (acyl-CoA Synthetase Short-chain Family Member 1), which has been associated with body weight, food intake, post-natal growth rate and susceptibility to weight loss among others (Liu et al., 2017).

### Gene Enrichment Analysis

Our findings indicated that pathways associated with production and mechanisms of environmental adaptation, such as immune response, male reproduction, energy production and heat stress, may be under selection in Ugandan goat breeds. This is in agreement with findings in East African Short-horn Zebu cattle (Bahbahani et al., 2015, 2017, 2018), South African cattle (Makina et al., 2015), and indigenous goats in Morocco and Egypt (Benjelloun et al., 2015; Kim et al., 2016). Gene ontology analysis shows that multiple pathways are expressed in the Ugandan goat breeds, which may indicate an adaptation to varied environmental conditions. This is also confirmed by recent studies with indigenous Sudanese goats which similarly implicated several biological processes (Rahmatalla et al., 2017). The multiplicity in the number of candidate regions and genes detected in the present study confirms findings from livestock species in stressful environments (Kim et al., 2016, 2017; Mwacharo et al., 2017). These studies and the current one, reaffirm the fact that adaptation is generally a complex trait, involving several biological processes and quantitative trait loci with each contributing a small but cumulative effect to the overall phenotype.

Although, the results based on raw *p*-values yielded very interesting biological pathways which may be overrepresented, the results of the more stringent multiple testing corrections such as Bonferroni correction, were not significant. This may be attributed to the small sample size involved in this study. Nonetheless, these results provide a useful indication of mechanisms involved in environmental adaptation in the indigenous goat breeds.

### Genomic Inbreeding Based on ROH (*F*_ROH_)

In the absence of pedigree records, ROH may be useful to infer the level of inbreeding. Computing the proportion of an individual’s genome occurring as an ROH of particular length (e.g., >1, >2, or >4 Mb) provides information on the level of inbreeding relative to a population several generations ago (Curik et al., 2014; Forutan et al., 2018). At ROH threshold of >2 Mb, the indigenous breeds showed very low levels of genomic inbreeding, as compared to the higher inbreeding levels found in the exotic Boer (**Table 2**). The low genomic inbreeding level reported in this study is consistent with findings in Swiss goat breeds (Burren et al., 2016) and Barki goats (Kim et al., 2016). Genomic inbreeding based on ROH provides an accurate estimate of an individual’s autozygosity than pedigree based inbreeding due to either incomplete or non-existent pedigree information (Ferenčaković et al., 2013a,b; Forutan et al., 2018).

Runs of homozygosity usually emanate from identical haplotypes being transmitted from parents to offspring (Purfield et al., 2012; Iacolina et al., 2016). The frequency of their existence provides a clue on the demographic history and management of the population over time (Kirin et al., 2010; Ceballos et al., 2018). The mean sum of ROH segment coverage was generally higher for short ROHs than for long ROHs (**Figure 3**). However, Karamojong showed a higher average sum of ROH_>__16__Mb_. The distribution of ROH coverage reported in this study is in agreement with other studies in goats (Brito et al., 2017), sheep (Purfield et al., 2017), and cattle (Ferenčaković et al., 2013a; Mastrangelo et al., 2016), in which long ROH segments were found less frequently compared to shorter ones. Although the short ROH were more frequent in the genome of the indigenous goat breeds, their absolute contribution to the genome was substantially low (except in Kigezi goats) (**Supplementary Table S7**). This result is consistent with findings of Bosse et al., (2012), who reported that short ROH were abundant in the porcine genome, but contributed less to the genome as compared to large ROH ( > 5 Mb). This may be due to differences in selection events in the more recent or ancestral populations. However, the short ROH in the Boer and indigenous Kigezi goats contributed more to the absolute coverage of the genome by the SNPs. The higher proportion of ROH segments within the short ROH categories indicates a relatively larger contribution of distant inbreeding, whereas the higher coverage of long ROH observed in Karamojong suggests a larger effect of more recent inbreeding. Karamojong goats are reared under pastoral production systems and may be subject to selection of best performing males by their keepers. This coupled with smaller effective population size could be contributing to the high recent inbreeding observed. On the other hand, the Kigezi goats are isolated populations that have undergone limited more recent selection, which could explain the high frequency of short ROH segments attributed to more distant inbreeding. The longer stretches of ROH in the exotic Boer goats may be due to the stringent artificial selection for production traits on few selection candidates (narrow genetic base) and may thus explain the higher levels of genomic inbreeding. Longer stretches of ROH were also observed in exotic goat breeds when compared to Barki goats in Egypt (Kim et al., 2016). Generally, shorter ROH is associated with more ancient inbreeding, while longer ROH tend to show a more recent inbreeding (Browning and Browning, 2012, 2013).

Although there are limited quantitative trait loci in goats, our study provides a basis for future research in goat genomics of tropically adapted breeds. Using medium density SNPs, we could detect selection signatures associated with adaptation to tropical environmental conditions. With the release of the caprine 50K SNP chip (Tosser-Klopp et al., 2014), several efforts are underway including improvements in the annotation of the goat genome assembly (Bickhart et al., 2017). Arguably, these developments will change the landscape of genomic research in goats, allowing for inclusion of genomic evaluations in goat breeding programs. The integration of genomic information will undoubtedly lead to better management and sustainable utilization of genetic resources. The results of this study will advance our understanding of environmentally driven adaptation and its potential application in functional genomics and selective breeding as well as in design of management programs to conserve livestock genetic diversity to cope with the current and future predicted effects of climate change.

## Conclusion

Using genome-wide SNP data, we investigated for the first-time selection signatures in Ugandan goat breeds that may be shaping their adaptation to varied environmental conditions. The study identified several putative genomic regions and genes in Ugandan goat populations, which may be underlying adaptation to local environmental conditions such as heat tolerance, disease and parasite resistance, and production traits. Generally, non-classical sweeps with limited overlap were observed which is typical of complex traits.

In the absence of pedigree data, genomic information through ROH provides a useful tool for quantifying the level of genomic inbreeding in the populations.

The study provides a foundation for detailed analysis of the identified putative selection signatures in the goat genome particularly of the tropically adapted breeds and provides an avenue for a well-structured breed improvement.

## Data Accessibility

Data from samples used in the present study are available from the Zenodo Digital Repository: https://doi.org/10.5281/zenodo.1184716.

## Ethics Statement

The animals sampled specifically for this study had their processes evaluated and approved by the Ethics Committee of Uganda National Council of Science and Technology (UNCST; SBLS/REC/15/131).

## Author Contributions

RO, MU, and MB conceived the study. RO drafted the manuscript. HD, MU, LB, and RO participated in the data analysis. MG, EK, RC, and MB supervised the study. All the authors read and approved the manuscript.

## Conflict of Interest Statement

The authors declare that the research was conducted in the absence of any commercial or financial relationships that could be construed as a potential conflict of interest.

## Footnotes

1 www.ncbi.nlm.nih.gov
