# Supplementary material for: Genome-Wide Characterization of Selection Signatures and Runs of Homozygosity in Ugandan Goat Breeds
Source: Front Genet. 2018 Aug 14;9:318. doi: 10.3389/fgene.2018.00318 (PMC6102322; doi:10.3389/fgene.2018.00318)
Supplement: Supplementary file 4 [file Table_4.DOCX]

**Table S4│**Overlapping putative regions of selection and genes identified by *F*_ST_ and hapFLK

| **Region** | **CHI** | **Region (Mb)** | **Breed** | **Genes** |
| --- | --- | --- | --- | --- |
| 1 | 6 | 4.408 – 5.018 | SEB | *PRDM5* |
| 2 | 6 | 8.162 – 8.423 | SEB | *LOC102173273* |
| 3 | 6 | 0.005 – 0.282 | SEA | *LOC102177053, LOC102190212, LOC102190503, LOC102190773, LOC102191032* |
| 4 | 6 | 15.380 – 15.641 | SEA | *ELOVL6, LOC102169085* |
| 5 | 6 | 8.212 – 8.614 | MUB | *LOC102173273* |
| 6 | 6 | 0.231 – 0.642 | KIG | *APELA, LOC102190212, LOC102190503, LOC102190773, LOC106503885, LOC102191032, LOC102191320, LOC102191601, LOC102168249, LOC102168527* |
| 7 |  | 8.244 – 9.011 | KIG | *TRAM1L1, LOC102173273* |
| 8 |  | 13.629 – 13.902 | KIG | *AP1AR, C6H4orf32* |
| 9 | 6 | 4.231 – 4.396 | KAR | *QRFPR, LOC108636256, LOC106502167* |
| 10 | 13 | 60.489 – 60.747 | SEB | *ID1, LOC102179296, BCL2L1, TPX2, MYLK2, LOC108637408, TTLL9, FOXS1, DUSP15, LOC102183156* |
| 11 | 13 | 59.093 – 59.482 | KIG | *SDCBP2, SNPH, RAD21L1, C13H20orf202, TMEM74B, PSMF1, RSPO4, ANGPT4, LOC102173205* |
| 12 | 13 | 63.523 – 63.986 | KIG | *PIGU, TP531NP2, NCOA6, ACSS2, GGT7, GSS, MYH7B, MIR499, TRP4AP, EDEM2, LOC102174673, LOC108637356, LOC102174406, LOC106502779* |
| 13 | 13 | 59.093 – 59.347 | KAR | *SDCBP2, SNPH, RAD21L1, TMEM74B, PSMF1, C13H20orf202, LOC102173205* |
| 14 | 15 | 21.318 – 21.533 | KAR | *MPPED2* |
| 15 | 15 | 17.890 – 18.068 | KIG | *ELF5, CAT, ABTB2* |
| 16 | 15 | 20. 574 – 20.879 | MUB | *ELF5, FMMP1L, DNAJC24, DCDC1* |
| 17 | 15 | 17.264 – 17.419 | SEA | *CD44* |
| 18 | 15 | 19.474 – 19.737 | SEB | *CCDC73, EIF3M, LOC108637654* |
| 19 | 16 | 40.710 – 41.244 | SEA | *DISP3, MTOR, UBIAD1, ANGPTL7, MASP2, SRM, EXOSC10, TARDBP, C16H1orf127, LOC106502956* |
| 20 |  | 43.006 – 43.669 | SEA | *CA6, ENO1, TRNAE-CUC, RERE, SLC45A1, LOC102176811* |
| 21 |  | 43.315 – 43.508 | MUB | *RERE, TRNAE-CUC, LOC102176811* |
| 22 |  | 40.594 – 41.215 | KAR | *MASP, TARDBP, MTOR, DISP3, C16H1orf127, UBIAD1, ANGPTL7, EXOSC10, SRM, LOC106502956* |

**KAR = Karamojong, KIG = Kigezi, MUB = Mubende, SEA = Small East African and SEB = Sebei**
