# Supplementary material for: Genome-Wide Characterization of Selection Signatures and Runs of Homozygosity in Ugandan Goat Breeds
Source: Front Genet. 2018 Aug 14;9:318. doi: 10.3389/fgene.2018.00318 (PMC6102322; doi:10.3389/fgene.2018.00318)
Supplement: Supplementary file 7 [file Table_7.DOCX]

**Table S7** Distribution of mean sum of ROH (Mb) according to ROH categories across six goat breeds

| **ROH category** | **Boer** | **Karamojong** | **Kigezi** | **Mubende** | **Small East African** | **Sebei** |
| --- | --- | --- | --- | --- | --- | --- |
| 2 - 4 | 102.734 | 4.180 | 13.384 | 6.903 | 7.277 | 2.653 |
| 4 - 8 | 116.917 | 5.729 | 6.995 | 4.809 | 4.924 | 1.867 |
| 8 -16 | 86.273 | 16.516 | 6.574 | 8.162 | 6.313 | 5.205 |
| > 16 | 33.673 | 33.604 | 4.437 | 9.741 | 10.577 | 9.604 |

**Table S8**: The percentage of chromosome residing in runs of homozygosity (ROH) per breed.

| **CHI** | **Boer** | **Karamojong** | **Kigezi** | **Mubende** | **Small East African** | | **Sebei** |
| --- | --- | --- | --- | --- | --- | --- | --- |
| 1 | 2.68 | 6.71 | 1.74 | 4.41 | 2.17 | 7.87 | |
| 2 | 3.81 | 3.07 | 3.24 | 4.58 | 1.81 | 1.50 | |
| 3 | 5.70 | 13.79 | 3.17 | 7.72 | 6.11 | 2.54 | |
| 4 | 5.11 | 11.31 | 4.32 | 2.34 | 5.92 | 2.45 | |
| 5 | 4.30 | 10.75 | 2.16 | 12.90 | 3.10 | 4.12 | |
| 6 | 4.29 | 13.63 | 3.96 | 8.87 | 5.22 | 8.76 | |
| 7 | 4.71 | 0.00 | 3.72 | 6.02 | 9.35 | 6.92 | |
| 8 | 4.97 | 14.53 | 4.24 | 15.02 | 5.42 | 11.01 | |
| 9 | 6.00 | 12.97 | 2.93 | 6.32 | 8.02 | 13.29 | |
| 10 | 5.36 | 10.39 | 2.96 | 2.19 | 3.15 | 22.22 | |
| 11 | 5.23 | 26.86 | 9.09 | 7.16 | 2.36 | 7.84 | |
| 12 | 7.30 | 8.61 | 3.33 | 5.60 | 3.71 | 13.29 | |
| 13 | 5.65 | 19.96 | 3.78 | 4.10 | 7.50 | 2.54 | |
| 14 | 6.16 | 22.94 | 3.04 | 4.38 | 21.92 | 19.88 | |
| 15 | 6.66 | 9.11 | 6.14 | 11.08 | 11.83 | 0.00 | |
| 16 | 6.79 | 2.64 | 4.57 | 5.58 | 5.34 | 0.00 | |
| 17 | 6.00 | 0.00 | 13.22 | 4.53 | 8.69 | 4.59 | |
| 18 | 8.54 | 6.58 | 5.50 | 4.53 | 4.15 | 8.83 | |
| 19 | 6.89 | 3.80 | 3.47 | 13.61 | 14.97 | 16.79 | |
| 20 | 7.15 | 0.00 | 13.36 | 3.87 | 11.19 | 21.78 | |
| 21 | 6.36 | 3.04 | 10.40 | 10.71 | 7.76 | 0.00 | |
| 22 | 7.28 | 16.44 | 6.37 | 7.40 | 17.35 | 0.00 | |
| 23 | 8.51 | 7.33 | 11.14 | 22.67 | 8.82 | 93.62 | |
| 24 | 8.84 | 0.00 | 14.06 | 18.36 | 0.00 | 4.72 | |
| 25 | 12.10 | 0.00 | 16.60 | 29.42 | 9.63 | 9.01 | |
| 26 | 8.64 | 34.42 | 6.47 | 7.45 | 0.00 | 24.33 | |
| 27 | 8.19 | 35.88 | 0.00 | 20.26 | 19.97 | 0.00 | |
| 28 | 10.81 | 0.00 | 14.00 | 0.00 | 27.84 | 4.80 | |
| 29 | 0.00 | 0.00 | 9.43 | 24.49 | 6.41 | 4.52 | |

**Table S9** Distribution of total number of ROHs across the chromosome in six goat breeds

| **CHI** | **Boer** | **Karamojong** | **Kigezi** | **Mubende** | **Small East African** | **Sebei** | **Totals** |
| --- | --- | --- | --- | --- | --- | --- | --- |
| 1 | 62 | 7 | 13 | 12 | 6 | 7 | 107 |
| 2 | 43 | 2 | 11 | 15 | 4 | 2 | 77 |
| 3 | 37 | 4 | 6 | 6 | 11 | 2 | 66 |
| 4 | 23 | 9 | 15 | 6 | 8 | 2 | 63 |
| 5 | 28 | 6 | 8 | 8 | 2 | 3 | 55 |
| 6 | 50 | 6 | 15 | 6 | 5 | 5 | 87 |
| 7 | 39 | 0 | 14 | 6 | 9 | 5 | 73 |
| 8 | 47 | 5 | 12 | 2 | 10 | 2 | 78 |
| 9 | 35 | 4 | 4 | 4 | 2 | 2 | 51 |
| 10 | 32 | 6 | 13 | 2 | 8 | 6 | 67 |
| 11 | 34 | 3 | 4 | 4 | 2 | 2 | 49 |
| 12 | 32 | 4 | 20 | 10 | 6 | 2 | 74 |
| 13 | 32 | 5 | 4 | 5 | 4 | 1 | 51 |
| 14 | 45 | 1 | 7 | 5 | 3 | 3 | 64 |
| 15 | 36 | 3 | 3 | 4 | 8 | 0 | 54 |
| 16 | 27 | 2 | 8 | 3 | 6 | 0 | 46 |
| 17 | 25 | 0 | 6 | 4 | 2 | 3 | 40 |
| 18 | 17 | 2 | 5 | 3 | 2 | 1 | 30 |
| 19 | 29 | 1 | 2 | 5 | 5 | 3 | 45 |
| 20 | 28 | 0 | 4 | 3 | 3 | 2 | 40 |
| 21 | 23 | 1 | 7 | 3 | 1 | 0 | 35 |
| 22 | 22 | 1 | 4 | 2 | 5 | 0 | 34 |
| 23 | 13 | 2 | 6 | 3 | 3 | 2 | 29 |
| 24 | 23 | 0 | 4 | 2 | 1 | 2 | 32 |
| 25 | 24 | 0 | 4 | 2 | 1 | 1 | 32 |
| 26 | 20 | 1 | 6 | 2 | 0 | 2 | 31 |
| 27 | 21 | 1 | 0 | 3 | 3 | 0 | 28 |
| 28 | 17 | 0 | 4 |  | 7 | 1 | 29 |
| 29 | 20 | 1 | 5 | 2 | 1 | 1 | 30 |
| **Totals** | **884** | **77** | **214** | **132** | **128** | **62** | **1497** |
