# Supplementary figures and images for: Genome-Wide Characterization of Selection Signatures and Runs of Homozygosity in Ugandan Goat Breeds
Source: Front Genet. 2018 Aug 14;9:318. doi: 10.3389/fgene.2018.00318 (PMC6102322; doi:10.3389/fgene.2018.00318)

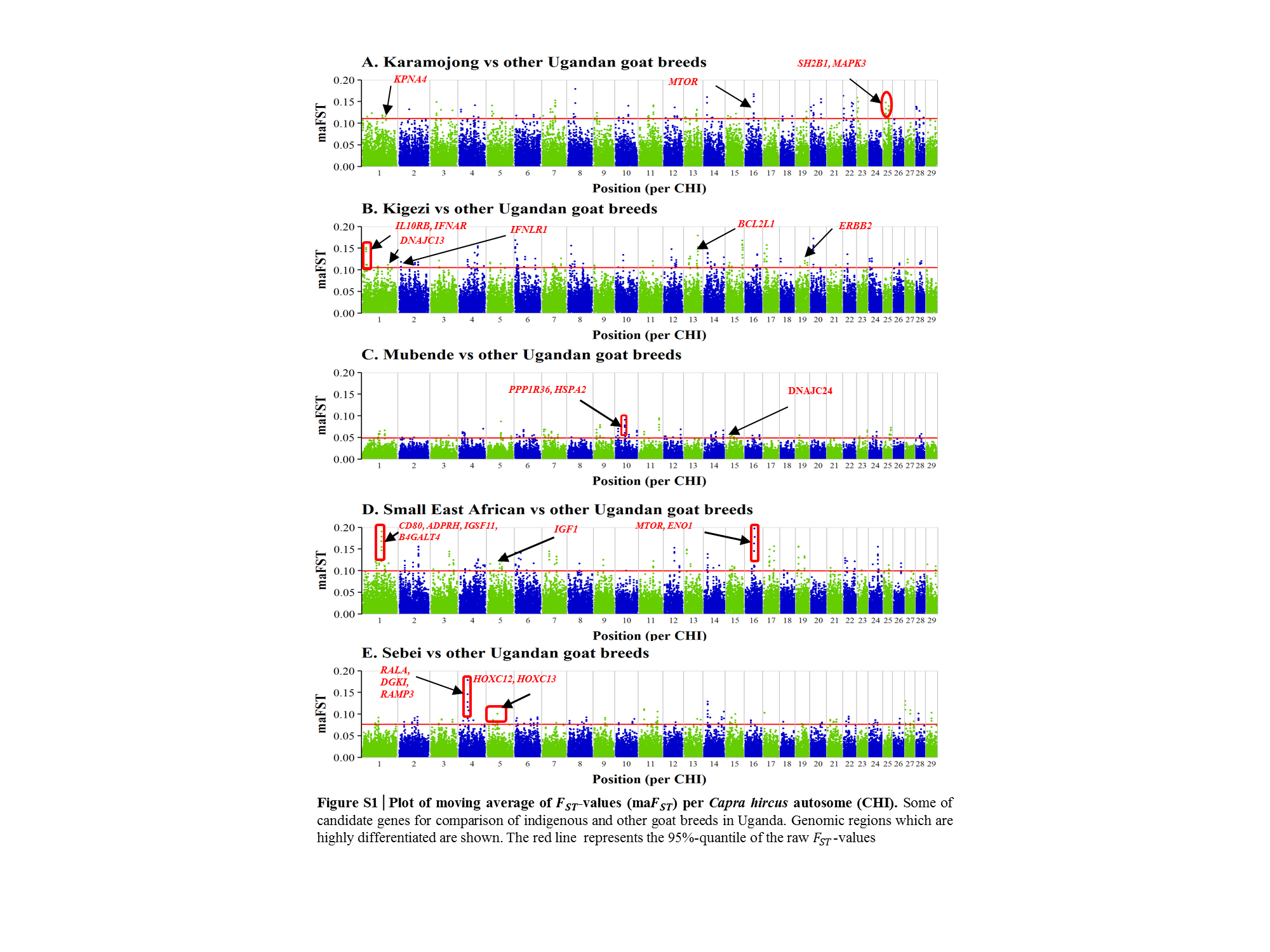

Supplement: Supplementary file 8 [file Image_1.TIF]

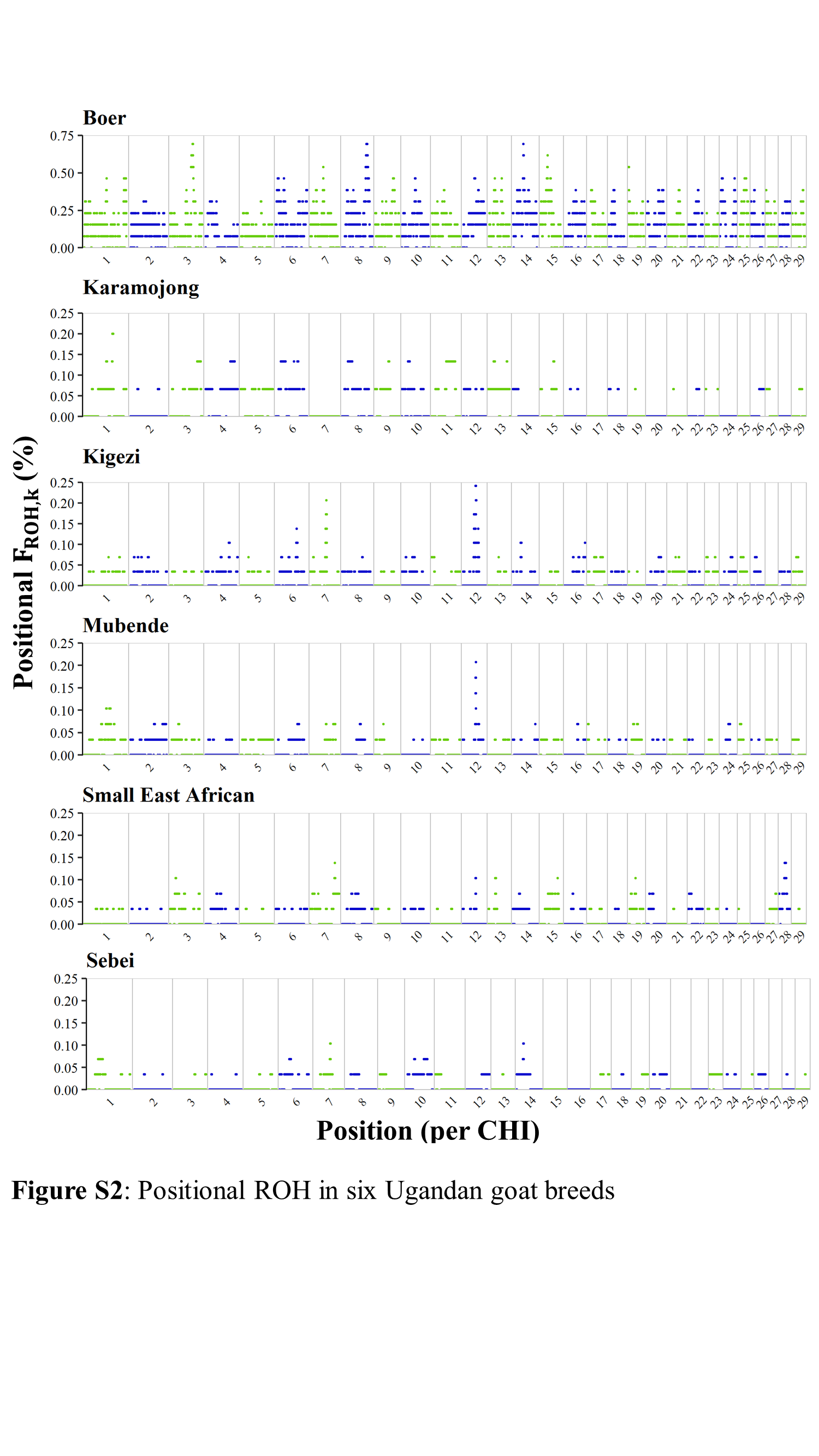

Supplement: Supplementary file 9 [file Image_2.tif]
